# Point-of-care ultrasound in German general practice: a qualitative study of opportunities and barriers

**DOI:** 10.1186/s12875-026-03585-y

**Published:** 2026-10-07

**Authors:** Mareike Ledigen, Anja Wollny, Christin Löffler, Til Uebel, Emil C Reisinger, Gregor Feldmeier

**Affiliations:** 1 Institute of General Practice, Rostock University Medical Centre, Rostock, Germany; 2 Institute of General Practice, University of Würzburg, Würzburg, Germany; 3 Division of Tropical Medicine and Infectious Diseases, Centre of Internal Medicine II, Rostock University Medical Centre, Rostock, Germany; 4 Rostock University Medical Centre Institute of General Practice, Postbox 100888, 18055 Rostock, Germany

**Keywords:** Conventional ultrasound, General practice, Point-of-care ultrasound (POCUS), Qualitative research, Rural areas

## Abstract

**Background:**

International studies show that point-of-care ultrasound (POCUS) is increasingly being used in primary care settings and can improve diagnostic decision-making. Particularly in the field of general practice, which is characterised by a lower prevalence of severe diseases, the use of POCUS may facilitate patient history-taking and diagnosis in a more targeted and efficient manner. This study aims to investigate how German general practitioners (GPs) perceive the clinical benefits, barriers, and implementation of conventional ultrasound use to date, as well as the benefit, barriers, and implementation requirements of POCUS in primary care.

**Methods:**

Between March and June 2023, two focus groups (FGs) were held at each of two locations (in Bavaria and Mecklenburg-Western Pomerania) as part of general practice quality circles. Using a convenience sample, each of the four FGs included between five and eight GPs. Semi-structured FGs were transcribed and anonymised. Using Mayring’s inductive content analysis method, two researchers analysed the FGs. The aim was to develop and identify new hypotheses and unexpected themes shedding light on both perspectives regarding the practical benefit and structural challenges of conventional ultrasound, as well as the potential of and barriers to the use of POCUS in general practice.

**Results:**

From the participants’ perspective, ultrasound has proven to be extremely relevant in everyday general practice. However, existing reimbursement frameworks and training requirements were perceived as significant barriers. The participants considered the implementation of POCUS in primary care as an opportunity to lower these barriers, improve diagnostic processes, and strengthen GPs’ skills.

**Conclusion:**

Our findings make it clear that the use of ultrasound currently depends largely on the individual commitment of GPs – in terms of the time involved and the financial resources required. However, it is also clear that POCUS could offer the potential to improve the conditions for the use of ultrasound in GP practices. In this way, the introduction of POCUS may improve diagnostic workflows and their accessibility in primary care.

**Supplementary Information:**

The online version contains supplementary material available at https://doi.org/10.1186/s12875-026-03585-y.

## Introduction

General practice operates in a low-prevalence setting. This presents the challenge of adopting a structured and resource-efficient approach to diagnosis [1, 2]. Within a stepwise diagnostic approach, it has been proven effective to use readily available, cost-effective procedures following the medical history, and subsequently, to supplement these with targeted, specialised, and more complex investigations. Point-of-care ultrasound (POCUS) can usefully complement primary care diagnostics with low-threshold imaging [3]. POCUS is an ultrasound examination performed directly at the point of patient care in a symptom-oriented manner to address a predefined clinical question [1, 3–5]. This focused approach is characteristic of the technique and contributes to a high level of diagnostic accuracy [6]. In general practice, there are only a few valid indications for its utilisation, e.g. ruling out thrombosis [1]. POCUS is not a simplified form of conventional ultrasound, but rather, represents an independent, targeted diagnostic procedure that may support clinical decision-making [7–10]. Whether a further sonographic examination is required depends on the extent to which the clinical question could be answered by POCUS [5]. The targeted use of POCUS is a (preceding) complement to comprehensive ultrasound diagnostics, with both procedures pursuing different diagnostic objectives. The current use of conventional ultrasound in the general practice setting is in line with the current requirements of the German Medical Association (BÄK) and the National Association of Statutory Health Insurance Physicians (KBV). While conventional ultrasound is an integral part of general practice training, obtaining billing authorisation poses a significant barrier: GPs are required to perform 400 supervised B-mode sonographies – a target that is challenging to achieve under current training conditions [1, 11]. In addition to the extensive training curriculum, a comprehensive diagnostic procedure is also required to formally bill for an ultrasound examination. This means that, for example, for a specific diagnostic question concerning the abdomen, the entire abdominal ultrasound examination must be carried out. This impacts not only the time needed but also the extent of the assessment comprising all organs as opposed to a genuinely problem-oriented diagnostic approach. In contrast to conventional ultrasound examinations, which are performed primarily by specialised physicians and only occasionally by primary care physicians, POCUS is intended to be performed directly by the treating primary care physicians themselves. The curricular requirements for teaching and learning POCUS in the general practice setting are a subject of controversy in the literature. Whilst Andersen and Jensen advocate for a curriculum lasting several months and involving mentoring [12], the European Federation of Societies for Ultrasound in Medicine and Biology (EFSUMB) recommends a 9-stage curriculum and a continuous learning process through participation in ongoing training programmes [13]. In contrast, a systematic review by a Danish working group highlights that focused POCUS training lasting just a few hours may be effective, although a wide variation in learning time was found ranging from 2 to 320 h [6]. There appears to be a consensus that participants have different and individual learning thresholds and, consequently, that the scope of competence-based learning also varies.

Even in rural and resource-poor regions, POCUS can enable a safe and timely assessment of clinical situations and improve primary care services. In these healthcare settings, implementation also benefits from the use of cost-effective, portable handheld ultrasound (HH-US) devices, which yield results comparable to those of conventional equipment in focused examinations [1, 14, 15]. There is evidence that the use of POCUS can reduce referrals and promote further treatment within general practice [7]. Thus, POCUS has become increasingly established internationally in primary care in recent years [5, 16]. Nevertheless, its direct practical use is not yet widespread. In a survey of general practitioners (GP), Andersen et al. addressed a possible misunderstanding in the direct comparison between conventional ultrasound examinations carried out by imaging specialists and POCUS performed by frontline clinicians. It has been shown that POCUS is a user-dependent examination and that, in the absence of sufficient knowledge, there is a risk of misinterpreting the ultrasound images which can lead to misdiagnoses, missed diagnoses, overdiagnoses and, consequently, under- or overtreatment. At the same time, they announced that there was insufficient evidence to support these concerns [17]. In Germany, wider application has primarily been found in emergency and pre-hospital medicine (e.g. within the framework of standardised protocols such as the FAST protocol [Focused Assessment with Sonography for Trauma]) [18]. However, the nationwide integration of POCUS into standard GP care remains limited. Systematic data on the prevalence, use, and evaluation of conventional ultrasound by GPs in Germany are currently unavailable [10]. Accordingly, it is important to identify influencing factors for the introduction of POCUS from the perspective of GPs. This study aims to investigate how German GPs perceive the clinical benefits, barriers, and implementation of conventional ultrasound use to date, as well as the utility, barriers, and implementation requirements of POCUS in primary care.

## Methods

### Study design and framework

This research utilised a qualitative content analysis design. These types of studies aim to dis-cover and understand the perspectives and points of view of the people involved to derive new hypotheses explaining the phenomena [19]. The analysis was based on the conceptual considerations regarding the evaluation of complex interventions according to the Framework of the UK Medical Research Council. Namely, as we view POCUS as a new intervention in primary care, our study corresponds to the feasibility study phase [20]. However, we wanted to capture GPs’ attitudes and perceptions as comprehensively and objectively as possible regarding the future use of POCUS. By using focus groups (FGs) and content analysis, we sought to generate new hypotheses and identify unexpected views on perspectives and structural and practical challenges [21]. The study adhered to the Standards for COnsolidated criteria for REporting Qualitative research (COREQ Checklist) reporting guidelines [22] (Additional file 1).

### Recruitment, sampling, and data collection

Between March and June 2023, two FGs were held at two locations in Bavaria (BY) and Mecklenburg-Western Pomerania (MV) (*n* = 4) as part of general practice quality circles. Quality circles are instruments of continuing medical education comprising moderated meetings of GPs to facilitate reflection on their own practice, professional exchange, and continuous improvement of the quality of care and patient safety. In Germany, the KBV established this structure as a quality assurance tool and coordinates the training and collaboration of those responsible [23]. Quality circles consist of a core group of GPs who meet regularly, usually in varying numbers. The topics of the continuing education sessions are chosen by the leaders and the group itself. Quality circles typically last between 1.5 and 2 h. Our FGs were conducted as part of quality circles on the same days. Each quality circle had several thematically distinct focal points that we were unaware of in advance. In addition, the participants did not know what the dedicated topic of our study was; they were simply told that we were interested in their perspectives on a topic relevant to primary care. The aim was to engage a convenience sample with a heterogeneous group of GPs. This way, participants were not limited to GPs interested in the topic. Recruitment for the quality circles took place by making personal contact with the group leaders, who then invited us to a meeting. The first FGs at both locations (BY 1 and MV 1) explored in which ways current ultrasound in the GP practices was carried out. The focus was on three key aspects: the everyday significance of ultrasound, the participants’ wishes and ideas regarding its ideal use, and the perceived obstacles and limitations in performing ultrasound examinations in a GP setting. The semi-structured question guide is shown in Table 1. During the second FG, participants were first introduced to the concept and potential applications of POCUS in general practice. The subsequent questions focused on basic opinions on implementation as well as imagined potential advantages and disadvantages in everyday general practice (BY 2 and MV 2). This question guide is shown in Table 2. The few questions were deliberately open-ended and, in some cases, phrased in a narrative style to explore topics and give participants the opportunity to discuss them. The sequenced questions were intended to promote an evaluation of current practice as well as to learn about attitudes towards the new approach. The four FGs were led by experienced and trained facilitators (GF, m, MD and AW, fem, PhD, both researchers at the Institute of General Practice, Rostock University Medical Centre) and supported by a co-facilitator (non-academic staff member of the same institute). Between five and eight GPs actively participated in the discussions, respectively (MV 1 = 8, MV 2 = 7, BY 1 = 5, BY 2 = 6). The participants were GPs who attended the quality circles (as a natural group). They were all practising family physicians between their mid-30s and mid-60s. No other sociodemographic data were collected. All participants in the initial quality circles were subsequently invited to the second quality circle. This allowed us to ensure that the composition of the groups remained similar. Between the first and second FG, there was one dropout in MV and a new person was added in BW.

**Table 1:** Focus group guide for conventional ultrasound in primary care

Type of question	Questions
Greeting and Introduction	Introduction of the moderators and explanation of the purpose of the study. Assurance of confidentiality and obtaining consent for recording.
Importance and Experiences	How important is ultrasound in your day-to-day practice?
	How do you perform it yourself?
Wishes	How would you like to use ultrasound if you were free to do so?
Challenges	What obstacles do you see to performing ultrasound? Do you see any limitations that might prevent you from performing it the way you’d like to?
Closing Remarks	Would you like to add anything else about your experiences? Is there anything else that hasn’t been addressed yet? Thank you very much for your time and for sharing your points of view. Your contributions are invaluable to this research.

**Table 2:** Focus group guide for POCUS in primary care

Type of question	Questions
Greeting and Introduction	Introduction of the moderators. Assurance of confidentiality and obtaining consent for recording. We’ll start with an introduction to POCUS using PowerPoint slides.
Ideas	How do you envision the introduction of this type of point-of-care ultrasound in primary care or in your practice? In your opinion, how would the use of POCUS affect the daily work of the next generation?
Benefits	Where do you see benefits?
Challenges	Where do you see challenges?
Closing Remarks	Would you like to add anything else from your experiences? Is there anything else that hasn’t been addressed yet? Thank you very much for your time and for sharing your points of view. Your contributions are invaluable to this research.

### Data analysis

**Fig. 1:**
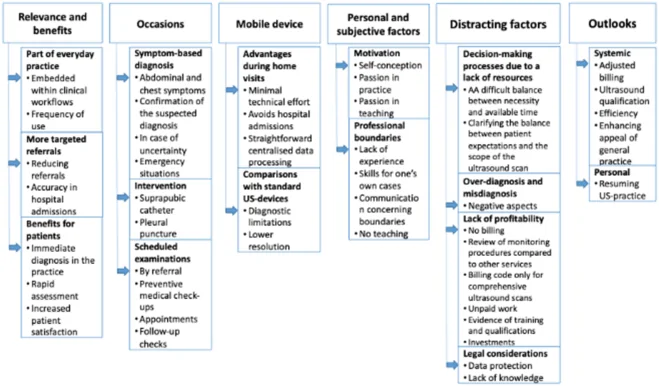
Coding tree ‘conventional ultrasound and POCUS in general practice’ (*BY1, MV1, BY2, MV2)* with main categories, categories, and subcategories

All FGs were audio-recorded with the participants’ consent and transcribed anonymously and verbatim by trained student assistants. In addition, the moderators produced a progress report summarising the course and content of each FG. The FGs took around 20 min on average. This study used conventional inductive content analysis following the methodology of Mayring [24]. That means that the categories were not predetermined but were developed from the material itself. Each FG was initially analysed to explore and understand themes and patterns of argumentation in the context of conventional ultrasound in general practice as well as the areas of application and the anticipated potentials and challenges of POCUS. ML (fem, researcher at the Institute of General Practice, Rostock University Medical Centre) and AW carefully reviewed the transcripts several times to examine the data thoroughly. Content analysis was performed by following the steps described by Mayring [25]. This included the creation of codes, subcategories, categories, and main categories. The coding was done independently by the two researchers ML and AW. After completing their individual analyses a consensus meeting took place to organise the data. Subsequently, the categories, subcategories, and main categories were merged together, and two codings (one for routine ultrasound practice and one for POCUS) were developed. After that, the coding trees were discussed and finalised within a larger, interdisciplinary working group. Numerous categories and subcategories relating to conventional ultrasound and POCUS appeared in both coding trees. Thus, it was decided to transfer the FG analysis into a single coding tree. The entire data set was then reviewed once more and adjusted where necessary. The result was an overarching coding tree comprising six main categories, sixteen categories, and forty-six subcategories (see Fig. 1). No specific software beyond Microsoft Word was used for this process.

## Results

The analyses shown in Fig. 1 are categorised as ‘Potentials of ultrasound’, ‘Challenges in current implementation’ and ‘Prospects arising from the introduction of POCUS in primary care settings’ for the purposes of a summary presentation. This allowed the research question to be answered in a targeted manner.

### Potentials of ultrasound

Across the FGs, many participants described using conventional ultrasound as part of their daily practice. One colleague explicated:

*‘Well*,* I couldn’t do without sonography. It’s performed every day nearly on a common basis… not commonly*,* you can’t really say that*,* but very*,* very often. For me*,* alongside the ECG – or*,* even before the ECG*,* if you like – it’s a very*,* very important diagnostic tool.’ (BY 1*,* TN 1)*.

Participants reflected on the limits of their expertise and described making conscious decisions about which examinations they perform themselves, whether to train junior colleagues in conventional ultrasound, and whether to undertake diagnostic procedures following referral from external colleagues. Participants described the ability to promptly confirm or rule out suspected diagnoses as an advantage of ultrasound. They suggested that this could enable more targeted referrals and hospital admissions. One participant explained:

*‘I actually do ultrasound to avoid having to send the patient somewhere else; that’s usually my motivation because my concern or experience is that someone has abdominal pain*,* you send them for an ultrasound*,* and you get back “the ultrasound is fine*,* let’s do a colonoscopy or gastroscopy”; then you send them for a gastroscopy*,* they come back saying “the gastroscopy is fine*,* now let’s do a colonoscopy” and then you get “the colonoscopy is fine*,* you can carry on”*,* and I think that cuts things short.’ (MV 1*,* TN 3)*.

Participants perceived that patients particularly benefited from the rapid clarification of their symptoms. One interviewee felt that a more precise diagnosis and more physical contact positively influence the doctor-patient relationship. Some participants reported using handheld ultrasound devices for an immediate diagnostic assessment even with immobile patients in care homes or in palliative care.

*‘Well*,* I can simply work much better out in the field; I can avoid hospital admissions. Particularly in the context of home visits*,* nursing homes*,* and such sites*,* its resolution is generally sufficient for the clinical questions at hand.’ (BY 2*,* TN1)*.

Despite lower resolution compared to stationary devices, participants considered the image quality of handheld devices sufficient for many general medical issues.

Participants mentioned a wide range of occasions for applying ultrasound including the exclusion of free fluid or an acute abdomen, pleural sonography, the exclusion of thrombosis, assessment of the urinary and biliary tracts, visualisation of the inferior vena cava to assess fluid balance, follow-up assessments of abdominal aortic aneurysms, and thyroid examinations. In the analysis, these indications for sonography could be categorised into symptom-related examinations (often in a POCUS-style manner), sonography-guided interventions, and planned examinations such as follow-ups and examinations on external referral.

Two distinct patterns of symptom-related ultrasound use emerged from the analysis. On the one hand, ultrasound was used for further diagnostic clarification in cases of unresolved clinical question. On the other hand, it was used as POCUS to answer a predefined clinical question, for example, to rule out deep vein thrombosis, urinary retention, or pleural effusion.

### Challenges of current implementation

The GPs reported that billing was only possible for extensive ultrasound examinations with full documentation. They also noted that even isolated findings, such as pleural effusions or an overfilled bladder, required documentation of a complete abdominal ultrasound to meet billing requirements, although the clinical question often only concerned the specific finding:

*‘The technology is supposed to confirm what I actually suspect based on the patient’s history and clinical presentation*,* and if I am then required to carry out a huge amount of unnecessary examinations just to be able to bill for it*,* that is virtually absurd.’ (MV 1*,* TN 8)*.

Further, they admitted to sometimes performing ultrasound examinations even when they could not bill for them. Otherwise the time required for documentation would exceed feasibility in routine practice. They described ultrasound as financially unattractive due to the combination of high investment costs and limited reimbursement opportunities. Participants also questioned the requirements for obtaining billing authorisation from the Ultrasound Commission of the Association of Statutory Health Insurance Physicians. In particular, they criticised the requirement for overwhelming documentation of examinations during training:

*‘When I see now what a time-consuming factor it is when a doctor or a colleague has to be scheduled because – I don’t know – she’s occupied with the patient for at least 20 minutes*,* even though perhaps only the gallbladder is of interest*,* but so that we can bill for it and it’s recognised for training purposes*,* then in that case she has to go through all these other things as well – so I think we’d use it much more frequently if it were possible to just focus on the bladder if you’re really only interested in the bladder*,* or the kidneys*,* or something like that*,* as an additional option to the overall picture.’ (MV 2*,* TN 5)*.

The GPs also expressed dissatisfaction with the spot-check reviews of billed ultrasound examinations conducted by the Ultrasound Commission of the Association of Statutory Health Insurance Physicians.

*‘Yes*,* but that’s the reason why I don’t bill for it […] because I don’t feel like having angles dictated to me*,* being told that wasn’t done properly*,* because for the clinical question I’m dealing within practice*,* it’s not relevant whether the kidney is consistently imaged in all planes – that’s the main reason for me: these tedious checks*,* having to sit there and pick out my images to submit to the Commission – I’m not doing that.’ (MV 2*,* TN 2)*.

Participants reported that billed ultrasound examinations were subject to review despite their billing authorisation and extensive experience. They noted that comparable review procedures did not apply to other diagnostic procedures, such as ECGs or medical histories. Some participants stated that they had stopped billing for ultrasound examinations to avoid time-consuming review procedures and potential complaints.

### Prospects arising from the introduction of POCUS in primary care settings

In the discussion on the implementation of POCUS in general practice, the participants developed a variety of perspectives. At a systemic level, structural and economic considerations were a recurrent topic:

*‘It would certainly make sense if*,* for the questions that arise in general practice – let’s say*,* is the bladder full or not – you could just put the transducer on and then be reimbursed for that without having to examine half the abdomen because then I could also make the decision more quickly.’ (MV 1*,* Participant 3)*.

Participants discussed POCUS as a potential framework for the reimbursement of ultrasound in general practice, either through a dedicated billing item or within GP-centred care programmes (Hausarztzentrierte Versorgung, HZV GP-centred care). They also reflected on the possibility of developing ultrasound training and accreditation criteria that better reflect the requirements of general practice. For example, they considered the recognition of ultrasound skills acquired during medical training and clinical practice. Further ideas included low-threshold continuing professional development programmes explicitly tailored to the specific requirements of general practice.

Moreover, participants discussed possible efficiency-related benefits of POCUS. They de-scribed opportunities to reduce costs, improve access through shared equipment, and delegate selected focused ultrasound examinations to trained non- physician personnel and thus address limited physician resources.

On a personal level, one participant explained that the concept of primary care POCUS prompted the idea of returning to ultrasound after a long break and refreshing diagnostic skills in this area:

*‘I’d really love to do it*,* but not with one of those €25*,*000 machines […]. That’s not how I picture it at all – not booking everyone in for an ultrasound at lunchtime*,* but really asking: can I sort this out here and now*,* or do I need to send the patient elsewhere? But actually*,* I wouldn’t go into debt to do that now. I would actually be very interested in doing further training that isn’t completely comprehensive – so not 400 images.’ (MV 2*,* TN3)*.

In this context, the participant linked interest in further ultrasound training to practical considerations, including the scope of training and the conditions required for implementing POCUS in practice.

## Discussion

### Summary of main findings and comparison with existing literature

The results showed that conventional ultrasound plays an important role in primary care in Germany. It is used both in acute situations for rapid assessment and specifically for further diagnostic evaluation (in a POCUS-like fashion), e.g. to specifically rule out thrombosis, urinary retention, or a pleural effusion. In some cases, abdominal ultrasound is also used in the context of POCUS. During the FG discussions, the participating GPs addressed many aspects that have also been documented or discussed in the international literature. For example, POCUS is increasingly recognized internationally as an efficient diagnostic tool in primary care [3, 6], and it is believed that POCUS can contribute to optimising treatment processes in primary care. This is consistent with the assumptions of the participating GPs. The areas of application identified in our discussion show clear similarities to the POCUS protocols used in the studies by Lo et al. and Colli et al. [10, 14] Studies have also confirmed the participants’ key points regarding device technology and image quality: they rated the image quality of handheld ultrasound devices as sufficient and helpful for specific clinical questions when compared to stationary devices [14, 15]. It was further noted that POCUS is not only diagnostically helpful but also promotes job satisfaction and enhances professional development. This is consistent with the findings of Kornelsen et al. [9], who identified a correlation between POCUS, increased self-confidence, and higher job satisfaction.

At the same time, the discussions highlighted significant structural barriers standing in the way of broader implementation of POCUS: existing regulations concerning the qualification, performance, and billing of conventional ultrasound examinations are considered unsuitable for the relevant clinical scenarios, particularly in low-prevalence settings. In a qualitative study, also conducted with GPs on the topic of POCUS, Andersen et al. described obstacles to the learning process, particularly those related to a lack of supervision, guidance, and sufficient opportunities for practice [26]. Ceriani et al. also highlighted a critical attitude toward POCUS in this context. Here attention is drawn to patient safety in the context of improper application, interpretation, and clinical integration of the results. This issue could perhaps be addressed through a significant expansion of training programmes [27]. To date, most studies and findings have originated from clinical inpatient settings and can only be applied to general practice to a limited extent [28, 29]. 

Our study emphasised that, to date, the use of ultrasound has depended strongly on the individual commitment of primary care physicians, both in terms of time and financial resources. However, participants claimed that POCUS could have the potential to improve the conditions for ultrasound use in primary care practices.

### Strengths and limitations

The study adopted an exploratory approach. The research examined the hitherto largely unexplored area of conventional ultrasound and POCUS use in general practice. The analysis was purely descriptive in nature, and it is not possible to conclusively assess whether the four FGs were sufficient for a comprehensive analysis of the topic. However, in all FGs, no new pivotal issues were raised towards the end, which could indicate thematic saturation.

The results of this study must be viewed in the light of further methodological limitations. Participation in the quality circles was voluntary, and the course leaders invited participants as part of the standard procedure. The natural groups within the quality circles had the advantage that, as the members know one another, the collective opinion had already been formed and was merely updated during the discussion [30]. This may explain the short duration of the FGs, as participants may have already covered the essentials and reached a consensus. It should be kept in mind that qualitative data collection in quality circles is not yet a widely used tool in Germany. Even though the group comprised participants who may have had little research experience, we were able to expect a high degree of authenticity and dynamic interaction due to the event’s framework. The fact that the participants were not familiar with our topic was an advantage. It meant that GPs who were not particularly interested in ultrasound also took part in our study. This could also lead to a stronger focus on practical application issues within the group. However, the fact that the participants knew each other well may also have been a disadvantage. The lack of anonymity and the resulting lack of confidentiality may have prevented critical issues from being raised. The survey was, however, deliberately conducted within the framework of quality circles, in which several different thematic focuses are always set. It can therefore be concluded that this approach enabled the involvement of a heterogeneous group of GPs who were not exclusively interested in a single predetermined topic.

Furthermore, it should be noted that the moderator – who had relevant prior experience with qualitative interviews – is a GP and the initiator of the study. Thus identification of topics relevant to practice and disadvantages could easily and authentically be facilitated during the interviews. To avoid any bias in the content analysis, the moderator was not part of the analysing team.

### Implications for practice and outlook

The results of this study support the assumption that the implementation of POCUS in general practice could be feasible and promising. This is consistent with international developments in primary care, where POCUS is increasingly recognised as a rational, mobile, and rapidly deployable tool for improving diagnostic-accuracy confidence and the quality of care. A standardised curriculum could help to ensure quality and reduce barriers to use, for example, in examination rooms or during home visits. Such approaches are currently being developed and tested or are already available [1, 10]. As the baby boomers reach an age where multimorbidity and frailty increase, a rising demand for medical care is expected [31]. Health policy addresses this by strengthening cross-sectoral, primary-care-centred care [32, 33]. GPs ensure continuity of care; coordinate the transition between outpatient and inpatient care for complex, polymorbid conditions; and manage the networking with specialist disciplines [34–36]. In this central role, POCUS in primary care can also support general outpatient palliative care in making well-informed and rapid decisions [37, 38]. International literature has shown that the targeted use of POCUS in primary care can help close gaps in care [9, 16, 39, 40]. The next steps should involve exploring suitable applications. In addition to structural improvements, the discussions also highlighted the importance of systematically integrating POCUS into medical education, continuing medical education, and professional development. This assessment is in line with the official statement of the World Organization of National Colleges, Academies and Academic Associations of General Practitioners and Family Physicians (WONCA), which recommends that all GPs receive curriculum-based, target-group-specific POCUS training as part of their residency and continuing medical education – including adequate financial support for the use of POCUS in primary care [3]. 

Further studies are needed to enable a well-founded basis for the benefits of POCUS in general practice, with both economic and clinical implications being systematically analysed. Furthermore, the views of non-users, specialists, and policymakers should be addressed, identified, and analysed. Research should examine the extent to which the use of POCUS within the respective healthcare system helps reduce unnecessary referrals, costly imaging procedures, and hospital admissions. Equally relevant are the questions of whether POCUS has a positive impact on the course of severe diseases through early detection and whether it influences patient safety and satisfaction in general practice. In addition, further research is needed on training and quality assurance in the use of POCUS to examine economic and clinical aspects. Of central importance is the definition of a standardised basic examination for general practice, as well as the development of training programmes integrated into the curriculum that ensure safe and effective use. The introduction of POCUS could thus be one of several responses to the impending challenges facing the healthcare system.

## Conclusion

To date, there have been no systematic studies on the prevalence and use of conventional ultrasound in general practice in Germany. The present study helps shed light on this area and shows that conventional ultrasound is already widely used in general practice, some of it even being applied in the form of POCUS. However, it is often performed without adequate framework conditions for training, licensing, and reimbursement. The study provides valuable insights into the perception and application of POCUS from the perspective of GPs. It offers important guidance for future research and implementation strategies, e.g. regarding training, billing, and barriers to investment.

## Supplementary Information

Below is the link to the electronic supplementary material.

Supplementary Material 1

## Data Availability

The datasets used and/or analysed during the current study are available from the corresponding author on reasonable request.
